# Enhancing the Transparency–Temperature Trade‐Off Through Spectral Engineering and Radiative Cooling

**DOI:** 10.1002/smsc.70311

**Published:** 2026-05-28

**Authors:** Pharit Gridtayawong, Taweesak Kaewmanee, Wachara Benchaphanthawee, Varakorn Phiriyasas, Chattrarat Ponghiransmith, Worawut Rueangsawang, Chaowaphat Seriwattanachai, Patawee Sakata, Napong Tangwiroon, Thantham Jittham, Napan Phuphathanaphong, Phatratorn Wonganannont, Tansuda Pinpapat, Tanant Waritanant, Tatpong Tulyananda, Pongsakorn Kanjanaboos

**Affiliations:** ^1^ School of Materials Science and Innovation Faculty of Science Mahidol University Nakhon Pathom Thailand; ^2^ Center for Cooling and Energy‐Saving Materials Faculty of Science Mahidol University Nakhon Pathom Thailand; ^3^ School of Bioresources and Environmental Biology Faculty of Science Mahidol University Nakhon Pathom Thailand; ^4^ Department of Environmental Science and Technology Faculty of Environment and Resource Studies Mahidol University Nakhon Pathom Thailand; ^5^ School of Bioinnovation and Bio‐based Product Intelligence Faculty of Science Mahidol University Nakhon Pathom Thailand; ^6^ Center of Excellence for Innovation in Chemistry (PERCH‐CIC) Ministry of Higher Education, Science, Research and Innovation Bangkok Thailand

**Keywords:** greenhouse thermal management, passive daytime radiative cooling, spectral engineering, TiO_2_–PET multilayer films, transparency–cooling trade‐off

## Abstract

Excess solar heat gain limits greenhouse productivity in tropical climates, where conventional polymer covers accumulate thermal energy and allow near‐infrared (NIR) transmission. Here, we demonstrate a scalable multilayer greenhouse film that mitigates daytime heat stress through spectral management of solar radiation combined with radiative cooling. A TiO_2_‐embedded polyethylene terephthalate (PET) scattering layer is laminated with ultraviolet (UV)–IR selective films to prevent excess heat while allowing appropriate photosynthetically active radiation (PAR) transmission. Two configurations are designed to address crop‐dependent light requirements: a higher‐transmittance film (∼57% PAR) and a stronger heat‐rejection film (∼37% PAR). The multilayer structures suppress NIR transmission (up to 80–92% rejection), reduce UV exposure, and exhibit near‐unity emissivity within the 8–13 µm atmospheric window (ε¯ ≈ 0.99), enabling efficient radiative heat dissipation. Outdoor rooftop measurements under tropical sunlight demonstrate consistent daytime temperature reductions of 3–5°C compared with those of commercial greenhouse films; the PET‐based laminate also provides high mechanical robustness (69–92 MPa tensile strength). These results establish spectral engineering as a practical strategy to manage the transparency–cooling trade‐off for passive greenhouse cooling in hot climates.

## Graphical abstract

This study presents a scalable multilayer greenhouse film for radiative cooling that balances photosynthetically active radiation and cooling performance through spectral engineering via combining TiO_2_–polyethylene terephthalate scattering with ultraviolet–IR selective layers. Outdoor and greenhouse tests demonstrate significant plant canopy‐level temperature reduction, highlighting an energy‐efficient solution for greenhouse agriculture in tropical climates.

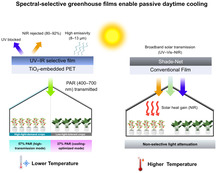

## 1 Introduction

Feeding a growing global population under accelerating climate change requires agricultural systems that remain productive despite intensifying heat stress from rising temperatures and extreme weather events, particularly since heatwaves are already reducing yields of major staple crops, with the most severe impacts occurring in tropical regions such as Southeast Asia, where food security and economic stability are tightly coupled to climate‐sensitive agriculture [1, 2, 3, 4]. Globally, anthropogenic climate change has reduced agricultural productivity by ≈21%, with disproportionately large losses in warmer regions [5, 6]. Controlled‐environment agriculture, especially greenhouse cultivation, offers a promising pathway to protect crops from climatic variability and enable stable, year‐round production [7, 8]. However, excessive solar heat gain in greenhouses operating in hot climates elevates plant respiration, suppresses photosynthesis, and increases water loss, driving heavy reliance on energy‐ and water‐intensive ventilation, cooling, and irrigation strategies [9, 10, 11]. Conventional approaches to mitigate greenhouse heat stress such as ventilation, evaporative cooling, and external shading can reduce internal temperatures but impose high operating costs that limit scalability in resource‐limited regions [12, 13, 14]. Among these strategies, shade nets are widely used to prevent greenhouse heat stress by reducing photosynthetically active radiation (PAR, 400–700 nm). However, their largely uniform attenuation reduces light intensity without spectral or angular control, often leading to suboptimal illumination at the plant canopy and lower photosynthetic efficiency, while also adding structural complexity through extra framing and seasonal installation [15, 16]. In hot tropical climates, greenhouse systems therefore rely on combinations of conventional polymer films (low‐density polyethylene (LDPE), polyethylene (PE)], and polyolefin (PO)) or diffuse commercial greenhouse films (DCFs) with inconvenient shade nets. More fundamentally, existing greenhouse films lack both passive radiative cooling capability and the mechanical robustness required for long‐term operation under severe tropical conditions, underscoring the need for an integrated, film‐based approach to thermal management.

Radiative cooling enables passive heat dissipation to outer space through the mid‐infrared (MIR; 8–13 µm) atmospheric transparency window [17, 18, 19, 20]. Passive daytime radiative cooling (PDRC) extends this concept by combining high solar reflectance with strong MIR emissivity, allowing subambient cooling even under direct sunlight [20, 21, 22, 23, 24]. In greenhouse applications, an ideal PDRC film would transmit PAR for plant growth while rejecting near‐infrared (NIR) solar heat and efficiently radiating internal thermal energy [22]. Recent advances in PDRC have demonstrated effective thermal regulation using multilayer photonic structures, nanoporous polymers, particle‐based composites, and transparent radiative cooling systems. For example, photonic and Bragg reflector‐based designs enable selective solar reflection while maintaining visible transparency [25, 26], while Janus‐type emitters have been proposed to mitigate heat accumulation in enclosed environments [27]. Scattering‐based polymer composites and coating systems have also achieved strong solar reflectance and high emissivity through optimized particle architectures, offering scalable and low‐cost fabrication routes [28].

However, these approaches exhibit fundamental limitations for greenhouse applications. Scattering‐based systems typically rely on broadband solar reflection and are often optically opaque, restricting PAR transmission. In contrast, photonic and transparent radiative cooling systems can achieve spectral selectivity but often depend on complex fabrication processes such as lithography or multilayer deposition, limiting large‐area manufacturability and practical deployment [29, 30, 31, 32, 33]. As a result, a gap remains between high‐performance radiative cooling materials and scalable, plant‐compatible greenhouse film solutions.

The adoption of PDRC in greenhouse agriculture remains limited due to the scarcity of polymer‐based films that simultaneously provide efficient radiative cooling and are compatible with large‐scale industrial extrusion or lamination processes. Nanoporous and composite polymer PDRC films based on poly(vinylidene fluoride), thermoplastic polyurethane, and polydimethylsiloxane have demonstrated substantial temperature reductions through enhanced solar reflection and thermal emission [33, 34, 35, 36, 37, 38, 39, 40, 41]; however, their reliance on laboratory‐scale fabrication techniques including phase separation, electrospinning, and template molding restricts scalability under commercial manufacturing conditions. In contrast, greenhouse films are predominantly produced from PE, ethylene–vinyl acetate, and polyethylene terephthalate (PET) due to their mechanical robustness, processability, and cost efficiency [42]. While these materials provide durability and high light transmission, they function largely as thermal barriers, promoting solar heat accumulation and limiting daytime temperature regulation [42, 43]. This gap between photonic performance and manufacturing scalability continues to hinder the practical implementation of radiative cooling strategies in agriculture.

**FIGURE 1:**
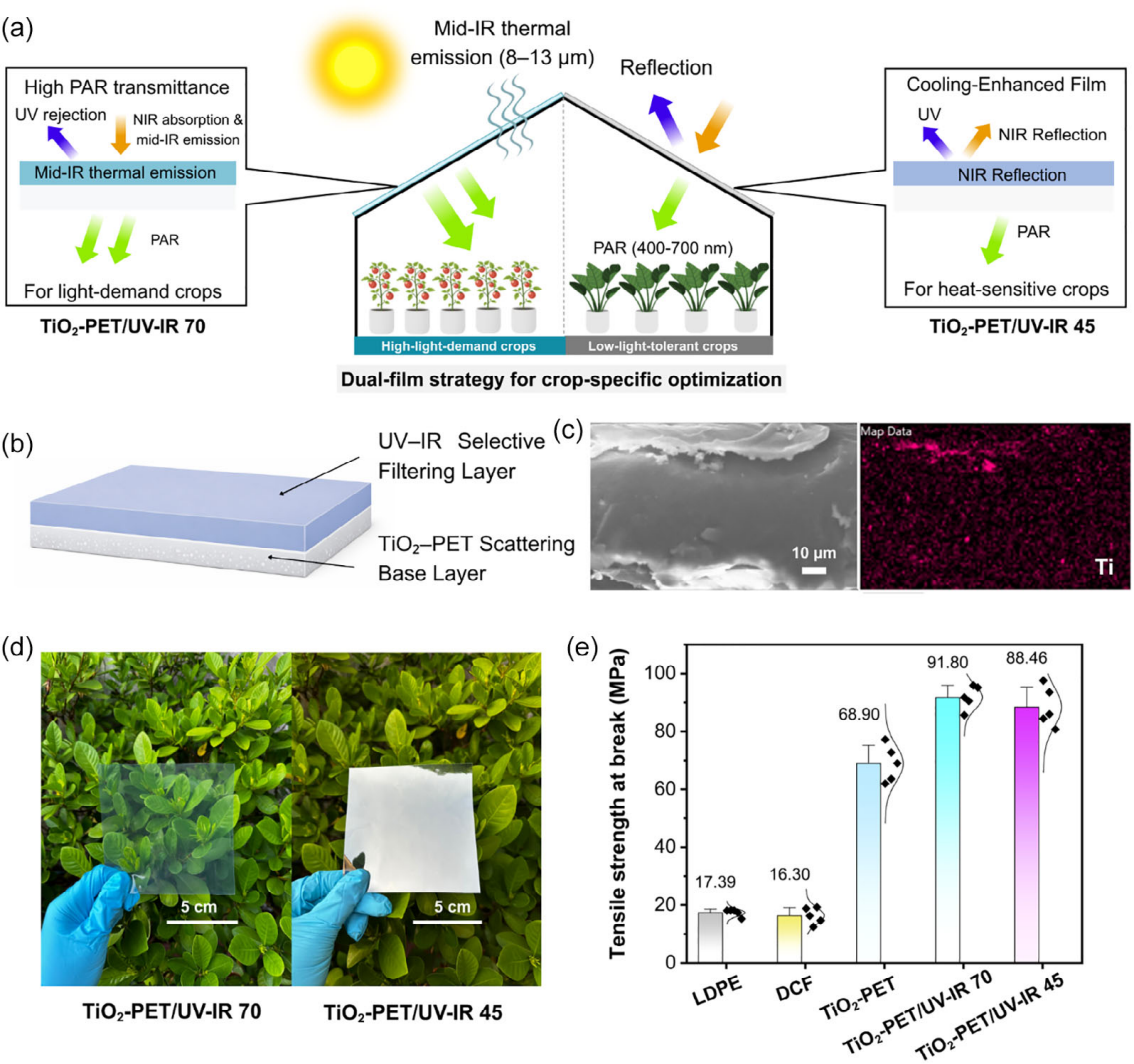
(a) Schematic illustration of a dual‐variant film strategy, resolving the trade‐off between PAR transmission and solar heat gain, (b) the construction of the multilayer film comprising a UV–IR selective filtering layer laminated on top of a TiO_2_–PET scattering base layer, where the UV–IR layer is the sky‐facing side of the structure, (c) cross‐sectional SEM image with corresponding energy‐dispersive X‐ray (EDX) elemental map to confirm the TiO_2_ dispersion within the PET matrix, (d) snapshot of two developed TiO_2_–PET/UV–IR greenhouse film variants at identical dimensions (10 × 10 cm), and (e) the plots of mechanical tensile strength of developed films compared to commercial greenhouse covers such as LDPE and DCF.

Here, we report a scalable polymer‐based greenhouse film that mitigates daytime heat stress in tropical climates by reframing greenhouse cooling as a problem of spectral energy management rather than maximal optical transparency. Under high solar irradiance, reduction of PAR is unavoidable; this work demonstrates how that reduction can be engineered to suppress solar thermal load while preserving biologically effective illumination. Instead of uniformly attenuating sunlight through shading, the proposed approach redistributes incoming solar energy spectrally and angularly within a single, mechanically robust film. The multilayer architecture consists of a TiO_2_–PET scattering base layer laminated with an ultraviolet (UV)–IR selective functional layer (Figure 1a,b), (commercially available UV–IR selective films, denoted as UV–IR 45 and UV–IR 70, corresponding to nominal visible transmittance of ≈45% and 70%, respectively), enabling independent control over solar‐band transmission and MIR thermal emission. The TiO_2_–PET layer introduces controlled light diffusion, improving canopy‐level light distribution and mitigating the physiological penalties typically associated with PAR reduction, while the UV–IR selective layers suppress NIR heat input through absorption‐ or reflection‐dominant pathways. Simultaneously, the laminated structure achieves near‐unity emissivity within the 8–13 µm atmospheric transparency window, enabling efficient radiative heat dissipation under outdoor conditions. To address crop‐dependent tolerance to PAR reduction, two film variants are intentionally designed: a higher‐transmittance configuration targeting light‐demanding crops and a lower‐transmittance configuration prioritizing thermal relief for heat‐sensitive crops. Built on a PET‐based laminate compatible with scalable extrusion and lamination processes, the films function as durable primary greenhouse coverings, eliminating reliance on shade nets or added structural complexity. Together, this work demonstrates that effective greenhouse cooling in hot climates does not require increased system complexity but a deeper integration of spectral control, plant‐compatible light management, and engineering durability within a deployable polymer film platform. The overall design strategy, multilayer architecture, structural characterization, and mechanical robustness of the developed greenhouse films are summarized in Figure 1.

Unlike conventional opaque radiative cooling coatings and transparent radiative cooling systems designed for glazing or esthetic applications, greenhouse operation imposes a fundamentally different constraint: thermal regulation must be achieved without fully sacrificing plant‐relevant photon transmission. The novelty of this work lies in formulating greenhouse cooling as a problem of spectral energy management implemented through a scalable polymer film architecture. The proposed TiO_2_–PET/UV–IR laminate integrates three key functions: (i) diffuse transmission of PAR, (ii) selective suppression of heat‐driving spectral bands (UV–NIR), and (iii) near‐unity emissivity in the MIR atmospheric window for radiative heat dissipation. By combining these functions within an extrusion‐compatible polymer laminate, this work addresses the transparency–temperature trade‐off under practical agricultural constraints, enabling deployment as a primary greenhouse covering rather than a supplementary optical layer. To clearly distinguish this approach from existing radiative cooling systems, Table 1 summarizes representative strategies in terms of materials platform, optical mechanism, fabrication, and application scope.

**TABLE 1:** Comparison of representative radiative cooling systems and the present work in terms of materials platform, optical strategy, scalability, and application constraints.

Study	Materials platform	Optical/thermal strategy	PAR/visible transmittance	MIR property	Fabrication/scalability	Target application	Demonstrated in greenhouse	Key limitation
Mandal et al. [43]	Hierarchically porous polymer coating	Broadband solar reflection + thermal emission	Opaque (∼0%)	High emissivity	Scalable coating	Buildings/ roofs	No	Blocks PAR; unsuitable for plant growth
Li et al. [17]	Structured polymer nanofiber selective emitter	Solar reflection + selective MIR emission	Low (<40%)	Selective emissivity (8–13 µm)	Electrospinning	Cooling surfaces	No	Insufficient PAR
Shan et al. [34]	TPU aerogel composite	Scattering‐based reflection + MIR emission	Opaque (∼0%)	High emissivity	Scalable NSPS process	Films/textile coatings	No	Opaque; not suitable for plant applications
Ko et al. [25].	Multilayer transparent film	Spectrally selective reflection (dual‐reflector multilayer)	Moderate (∼50–70%)	High emissivity	Multilayer + microstructure fabrication	Windows	No	Not tested for plant growth
Park et al. [27]	Janus multilayer transparent film	Solar reflection + asymmetric emission + internal heat absorption	Moderate (∼55–60%)	High emissivity (top, AW) + broadband absorption/emission (bottom)	Multilayer thin‐film (DMD + Bragg reflector) fabrication	Enclosed systems (e.g., vehicles, buildings)	No	Not suitable for plant greenhouse applications
Yun et al. [28]	Microparticle coating	Broadband scattering	Opaque (∼0%)	High emissivity	Scalable coating	Buildings	No	Blocks PAR
Zou et al. [44]	Transparent radiative cooling film	PAR transmission + NIR reflection + MIR emission	∼69% PAR	High emissivity (8–13 µm)	Multilayer film	Greenhouse covering	Yes	Multilayer structure; condition‐dependent performance
Li et al. [45].	Photonic crystal/hydrogel multilayer	PAR‐selective transmission + MIR emission	∼70–77% (PAR bands only)	High emissivity	Complex multilayer nanofabrication	Plant cooling	Yes	Complex fabrication; limited spectral flexibility
Jiang et al. [46]	Polymer multilayer RC film	Selective PAR transmission (0.4–0.48, 0.6–0.7 µm) + green/NIR reflection + MIR emission	∼52–85% (λ‐dependent)	High emissivity	Multilayer coextrusion; scalable	Plant heat stress mitigation	Yes	Limited NIR reflectance; performance–scalability trade‐off
Tian et al. [47]	PE/Cs_0_._33_WO_3_ cover + cellulose RC mulch system	NIR absorption + high MIR transmission (cover) + solar reflection/MIR emission (mulch)	∼70% PAR	Cover: MIR transmittance; mulch: high emissivity	Film‐blown + biodegradable cellulose processing; scalable	Greenhouse passive cooling (sky–ground system)	Yes	Requires integrated cover–ground system; NIR absorption‐induced heating; system‐level complexity
Vallan et al. [48]	Paraffin–PVA thermochromic composite	Phase‐change‐induced refractive‐index mismatch → broadband light scattering (thermotropic modulation)	∼77% (transparent state)	Not MIR emissivity‐driven; no radiative cooling design	Solution‐processed doctor‐blade multilayer coating; scalable	Smart windows/greenhouse demonstration	Yes	Not spectrally optimized for radiative cooling
This work	TiO_2_–PET + UV–IR laminated film	Partial PAR transmission + NIR attenuation + MIR emission	37–57% (tunable)	High emissivity ( 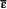 ≈ 0.99)	Extrusion + lamination	Greenhouse covering	Yes	Trade‐off between PAR transmission and cooling

*Note*: PAR transmittance values are estimated from reported spectral data over 400–700 nm (photosynthetically active radiation).

## 2 Results and Discussion

### 2.1 Optical Spectral Engineering in the Solar Band (PAR–NIR)

**FIGURE 2:**
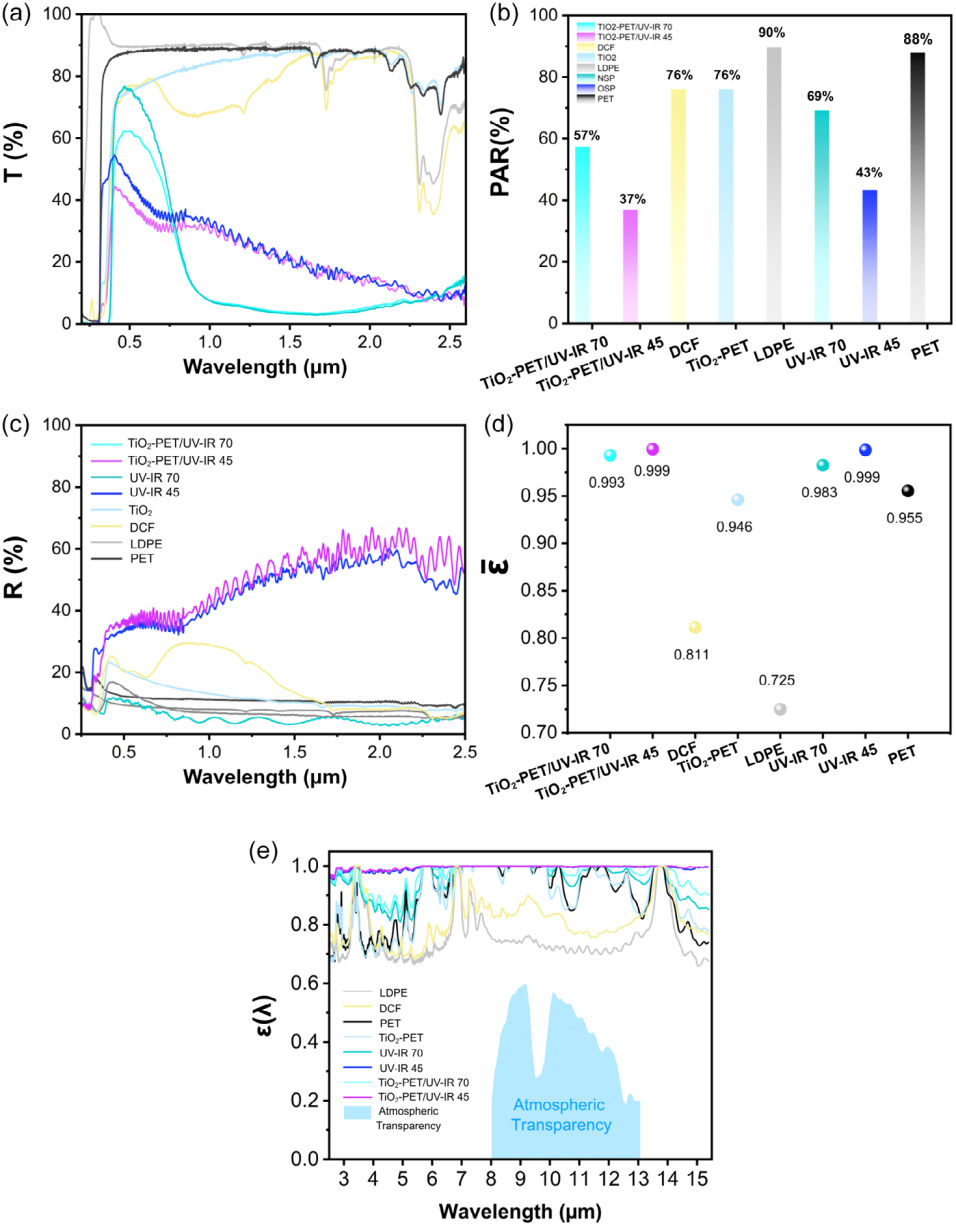
(a) UV–Vis–NIR and (b) PAR transmittance spectra of films in this work, (c) solar reflectance spectra in range of 0.3–2.5 µm of films in this work, and (d) blackbody‐weighted mid‐infrared emissivity and (e) spectral emissivity with atmospheric transparency in the range of 8–13 µm.

Building on the multilayer architecture illustrated in Figure 1, the solar‐band optical response (Figure 2a–c) illustrates how controlled scattering and spectral selectivity are used to manage the inherent trade‐off between PAR transmission and solar heat gain. Rather than maximizing optical transparency, PAR reduction is treated here as an intentional control variable for thermal regulation under high solar irradiance. Pristine PET exhibits high broadband transparency, transmitting 87.9% of PAR (Figure 2a,b); however, this high transparency is accompanied by substantial NIR transmission, which directly contributes to solar thermal loading. Introducing a low TiO_2_ loading into PET reduces PAR transmittance to 76.1% (Figure 2b), primarily due to wavelength‐dependent Mie scattering arising from the refractive‐index contrast between rutile TiO_2_ particles and the PET matrix. Laser scattering measurements (Figure S6), supported by scanning electron microscopy (SEM) observations (Figures S7–S8), reveal an effective particle size in the micrometer range (D50 ≈2.15 µm), indicating aggregation of primary nanoparticles into micron‐scale domains. This size regime is consistent with efficient wavelength‐dependent scattering across the visible–NIR range, as expected for dielectric scatterers of comparable dimensions. At this size and loading, scattering predominantly redistributes incident radiation with minimal absorption, reducing NIR transmission while preserving a functional PAR window. Importantly, the low TiO_2_ concentration mitigates particle crowding, preserving interparticle spacing and avoiding the reduction in scattering efficiency associated with multiple scattering and near‐field coupling effects at higher loadings [49]. As a result, the TiO_2_–PET film establishes an intermediate optical regime between full transparency and aggressive solar rejection, in which partial suppression of solar heat input is achieved through scattering‐induced redistribution rather than uniform attenuation. To address crop‐dependent light requirements, two commercially available UV–IR films with distinct thermal management strategies have been chosen to meet the specific light needs. The UV–IR 70 film suppresses NIR mainly through absorption and exhibits a low average NIR reflectance of around 4%, while maintaining a high PAR transmittance required for light‐demanding crops. On the other hand, the UV–IR 45 film uses a reflection‐based strategy to reject NIR enabling more aggressive solar heat rejection for shade‐tolerant crops (Figure 2c). When UV–IR 45 is laminated onto the scattering TiO_2_–PET substrate, the reflection‐dominated behavior of the UV–IR 45 film is further enhanced. The TiO_2_–PET/UV–IR 45 stack exhibits an increased average NIR reflectance of ∼54% across the 0.8–2.5 µm range, while achieving slightly lower NIR transmittance compared with the standalone UV–IR 45 film (Figure 2a,c). This change arises from the TiO_2_–PET layer, which causes increased optical path length and more internal reflections. In contrast, the TiO_2_–PET/UV–IR 70 couple remains absorption‐dominant, showing only marginal changes in NIR reflectance. These results show that absorption‐ and reflection‐dominant thermal management strategies can be selectively combined through multilayer spectral engineering. By integrating a scattering polymer base with UV–IR selective laminates, the system enables tunable transparency–cooling trade‐offs tailored to crop‐specific light demands, rather than relying on a single universal spectral solution.

### 2.2 MIR Emissivity and Radiative Cooling Potential

Beyond controlling solar heat input through spectral management in the solar band, effective greenhouse cooling also requires the dissipation of accumulated thermal energy through the MIR atmospheric transparency window (MIR, 8–13 μm). The spectral emissivity and blackbody‐weighted average emissivity of the investigated films within this range are shown in Figure 2d,e. The blackbody‐weighted average emissivity (ε¯) over the 8–13 μm atmospheric transparency window is used here as a quantitative metric for radiative cooling performance (see Methods for definition). Pristine PET exhibits high, though incomplete, MIR emissivity (ε¯ ≈ 0.96), arising from vibrational modes of the polymer matrix dominated by C=O stretching and C–O deformation [50, 51, 52]. Incorporating a low TiO_2_ loading into PET does not significantly alter this response (ε¯ ≈ 0.95), consistent with the role of TiO_2_ in this system as a solar‐band scattering medium rather than a MIR‐active absorber at low concentrations. As a result, the intrinsic emissivity of the polymer matrix remains the dominant contributor to thermal radiation.

In contrast, the laminated TiO_2_–PET/UV–IR structures exhibit near‐unity emissivity across the atmospheric window. The TiO_2_–PET/UV–IR 70 and TiO_2_–PET/UV–IR 45 stacks reach blackbody‐weighted emissivity of ε¯ ≈ 0.99 for both samples, significantly exceeding those of pristine PET and TiO_2_–PET. This enhancement arises not from oxide incorporation alone but from the multilayer optical architecture, which increases effective absorptivity in the MIR through multiple internal reflections and optical impedance matching [53]. According to Kirchhoff's law of thermal radiation, the enhanced absorptivity directly translates into increased emissivity, thereby strengthening radiative heat dissipation to the sky [54, 55]. By comparison, commercial LDPE and DCFs exhibit considerably lower MIR emissivities (ε¯ ≈ 0.73 and ε¯ ≈ 0.81, respectively), highlighting their limited capacity for radiative cooling despite widespread use in greenhouse applications. These results underscore a key design principle: while polymer chemistry establishes a baseline MIR response, multilayer spectral architecture governs radiative cooling effectiveness under practical operating conditions. When combined with intentional PAR reduction in the solar band, high MIR emissivity enables thermal energy to be efficiently rejected rather than retained, reinforcing the role of spectral engineering in managing the transparency–cooling trade‐off.

### 2.3 Outdoor Cooling Performance

**FIGURE 3:**
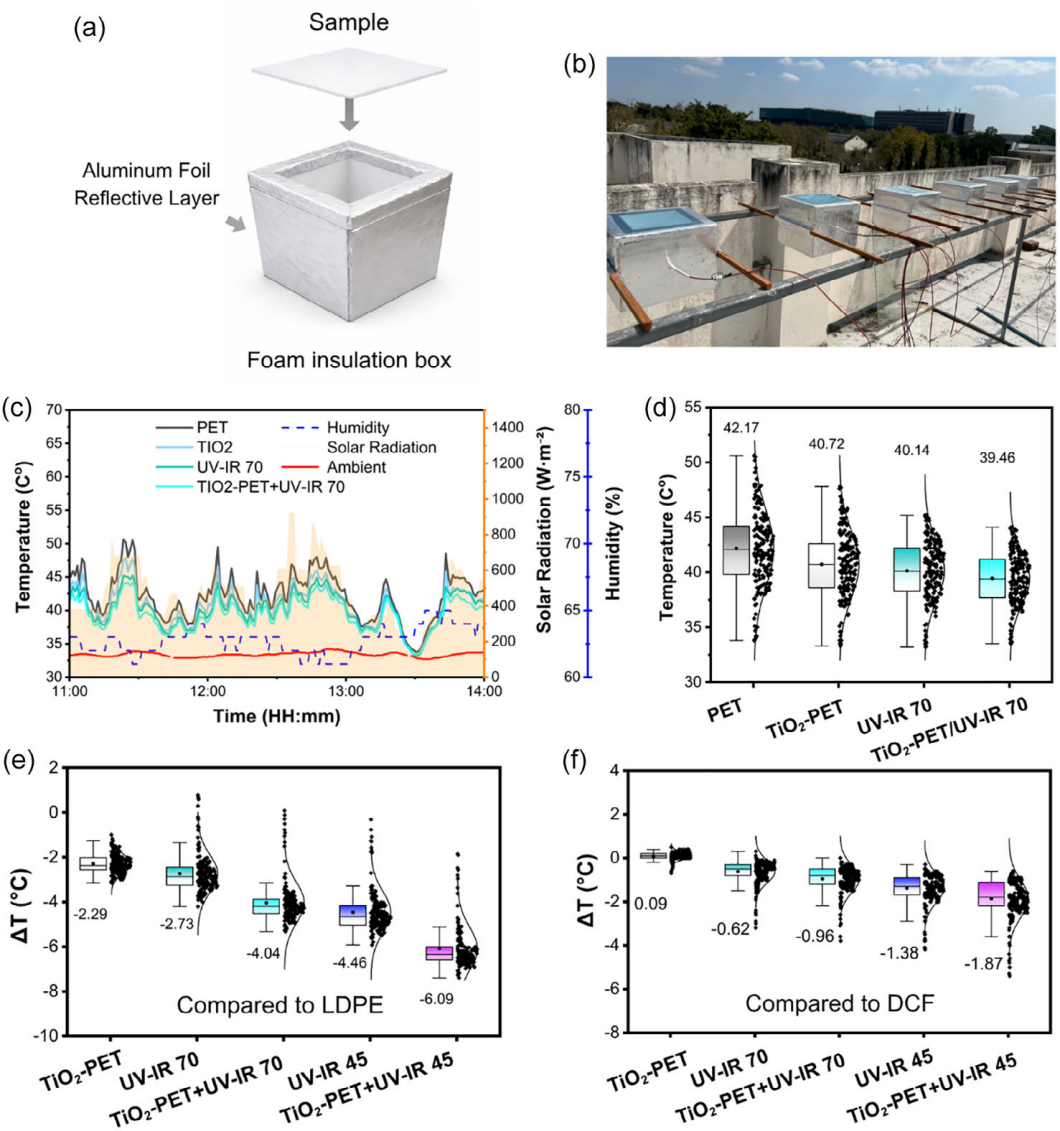
(a) Construction of the insulated test box; film samples were sealed over the top opening of an aluminum‐lined foam box (see Figure S4). (b) Photographs of the outdoor experimental setup under clear‐sky conditions. (c) Time‐resolved temperature profiles between 11:00 and 14:00 (local time) with solar irradiance and ambient humidity of films under clear‐sky conditions on 19^th^ July 2025. (d) Statistical temperature distribution of films on 19^th^ July 2025. (e) Temperature reduction (ΔT) of the developed films compared to LDPE, measured on 2^nd^ August 2025 under comparable outdoor conditions. (f) Temperature reduction (ΔT) of the developed films compared to diffuse IR‐cut commercial greenhouse film (DCF), measured on 24^th^ July 2025 under comparable outdoor conditions.

Outdoor rooftop measurements were conducted at the Faculty of Science, Mahidol University, Nakhon Pathom, Thailand, under clear‐sky conditions on 19^th^ July, 24^th^ July, and 2^nd^ August 2025 between 11:00 and 14:00 local time, to evaluate the thermal performance of the proposed films under realistic tropical solar conditions (Figure 3). Measurements for different samples were conducted on separate days under similar clear‐sky conditions within the same seasonal period. Ambient parameters, including solar irradiance and humidity, were continuously monitored to ensure consistency across measurements, and all data were analyzed within the same time window (11:00–14:00) to minimize the influence of diurnal variations. Foam‐box measurements were conducted during the tropical wet (monsoon) season (July–August 2025), whereas small‐scale greenhouse validation was performed during the summer season (April 2026). Under identical exposure, pristine PET exhibits the highest surface temperature throughout the measurement period, consistent with its high transmittance of both PAR and NIR radiation, which leads to substantial solar heat gain. Incorporating TiO_2_ into PET results in a consistent temperature reduction relative to pristine PET, reflecting partial suppression of forward‐propagating NIR photons through scattering while maintaining a functional PAR window. This intermediate cooling response is consistent with the solar‐band optical behavior discussed in Section 2.1. More pronounced cooling is observed for films incorporating UV–IR selective layers. The standalone UV–IR 70 film exhibits a clear temperature reduction relative to PET by limiting solar heat input primarily through absorption‐dominant NIR suppression. The strongest cooling performance is achieved by the TiO_2_–PET/UV–IR multilayer films, demonstrating the synergistic effect of combining controlled scattering with selective solar‐band rejection. Among these configurations, the TiO_2_–PET/UV–IR 45 film consistently exhibits the lowest average temperature under peak solar irradiance (Figure S5). This enhanced cooling arises from reflection‐dominant NIR rejection combined with near‐unity MIR emissivity, which together reduce solar heat gain and promote radiative heat dissipation through the atmospheric transparency window, as widely reported in outdoor radiative cooling studies [18]. Importantly, the relative temperature rankings remain consistent across the measurement period despite variations in solar irradiance and ambient humidity (Figure 3c,d), indicating that the observed cooling trends are governed primarily by intrinsic spectral design rather than transient environmental fluctuations.

### 2.4 Benchmarking and Transparency–Cooling Trade‐Offs

Benchmarking against commercial greenhouse coverings provides a quantitative assessment of cooling performance under tropical outdoor conditions (Figure 3e,f). Across all reference baselines, increasing daytime temperature reduction (ΔT) is consistently associated with decreasing PAR transmission, confirming that suppression of solar heat input under strong sunlight inevitably incurs optical loss. Relative to LDPE and DCFs, progressively stronger cooling is observed from TiO_2_–PET to the TiO_2_–PET/UV–IR multilayer films, with the TiO_2_–PET/UV–IR 45 configuration achieving the largest temperature reductions (up to 5–6°C, depending on the reference baseline) while maintaining a moderate PAR transmittance of 37%. Importantly, these trends cannot be explained by PAR reduction alone. TiO_2_–PET exhibits only a modest decrease in PAR transmission (∼76%) yet delivers a measurable temperature reduction of ≈1–2°C relative to commercial baselines. This behavior indicates that partial suppression of solar heat gain through scattering‐induced redistribution of incident radiation can deliver meaningful thermal benefits without aggressive broadband attenuation, consistent with prior reports on diffuse and scattering‐based greenhouse coverings [56, 57]. As shown in Figure 2a and c, the multilayer TiO_2_–PET/UV–IR films enhance cooling through spectral energy management rather than broadband shading. The coating rejects heat‐carrying radiation while maintaining plant‐useful light. TiO_2_–PET/UV–IR 45 maintains a PAR transmittance of 37% while suppressing NIR transmission to 19.8% (80.2% rejection) and reducing UV transmission to 7.1%. Similarly, TiO_2_–PET/UV–IR 70 retains 57.3% PAR while blocking 91.8% of NIR and transmitting only 2.7% UV. In contrast, conventional greenhouse LDPE films transmit ∼83% UV and ∼84% NIR, permitting nonphotosynthetic solar energy to enter the enclosure where it is absorbed by internal mass and re‐emitted as long‐wave heat. Elevated plant canopy temperature and UV exposure induce stomatal closure, accelerate photorespiration, and promote photoinhibition of PSII, thereby reducing carbon assimilation despite similar photon availability [10, 58]. Beyond solar filtering, the multilayer films exhibit near‐unity emissivity within the atmospheric window (ε¯ ≈ 0.99, 8–13 µm; Figure 2d), substantially higher than LDPE (ε¯ ≈ 0.73; Figure 2d), and DCF (ε¯ ≈ 0.81; Figure 2d), enabling continuous radiative heat dissipation to the sky. Selective rejection of UV–NIR radiation combined with efficient mid‐IR emission lowers net heat flux without proportional PAR loss. Conventional shade nets reduce all wavelengths equally, lowering UV, PAR, and NIR to 37% or 57% of sunlight. In contrast, the spectrally engineered films keep PAR while removing heat‐carrying radiation and enhancing mid‐IR emission, allowing more radiative cooling.

### 2.5 Application‐Level Validation in a Small‐Scale Greenhouse

**FIGURE 4:**
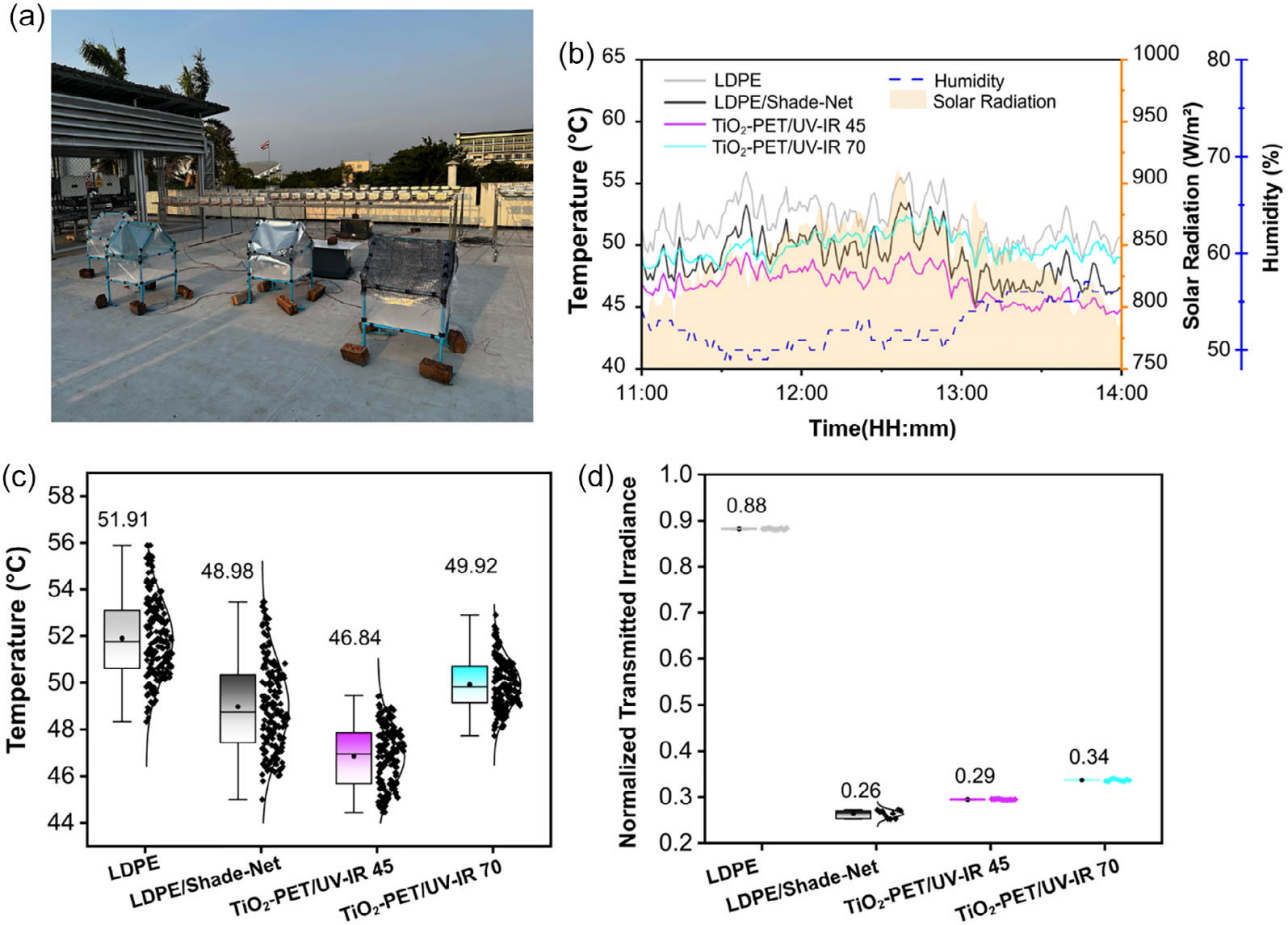
(a) Snapshot of the greenhouse units used for outdoor testing. (b) Temperature profiles between 11:00 and 14:00 with solar irradiance and ambient humidity of films under clear‐sky conditions during the hot season of Thailand (April, 2026). (c) Statistical distribution of temperature between 11:00 and 14:00. (d) Normalized transmitted irradiance for each configuration.

To assess practical performance, outdoor experiments were conducted using identical polyvinyl chloride (PVC)‐framed greenhouse units with the covering material as the only variable (Figure 4a). Geometry, orientation, ground conditions, and ventilation were kept consistent. Plant canopy‐level temperature (i.e., at the height of the plant leaves) was selected as the primary metric, as it directly reflects the thermal environment experienced by plants. Under a solar irradiance of 720–730 W m^−2,^ LDPE exhibits the highest canopy temperature, followed by TiO_2_–PET/UV–IR 70 and LDPE combined with a shade net (nominally rated at 60% shading by the manufacturer), while TiO_2_–PET/UV–IR 45 consistently yields the lowest temperature (Figure 4c). Relative to LDPE, TiO_2_–PET/UV–IR 45 reduces the canopy temperature by ∼5.1°C, whereas LDPE combined with a shade net achieves a smaller reduction of ∼2.1°C. Prior LDPE‐only greenhouse calibration confirms consistent baseline thermal behavior across all units with low inter‐unit variation (σ ≈ 0.17°C; Figure S11). To decouple the effect of total solar transmission from spectral design, the transmitted irradiance near the canopy was quantified under outdoor conditions (Figure 4d). The corresponding outdoor irradiance measurements prior to normalization are provided in Figure S12, confirming that the incident solar input across configurations is comparable under real‐world conditions. TiO_2_–PET/UV–IR 45 and LDPE combined with a shade net exhibit comparable normalized transmitted irradiance (∼0.29 and ∼0.26, respectively), consistent with controlled 1 Sun indoor measurements (Figure S13), confirming that differences in thermal performance cannot be attributed solely to variations in total transmitted irradiance. Despite similar transmitted irradiance levels, TiO_2_–PET/UV–IR 45 maintains a substantially lower canopy temperature. UV/NIR blocking as shown in Figure 2 enables more effective thermal regulation per unit of transmitted irradiance compared to conventional shading, highlighting the advantage of selective spectral management over broadband attenuation strategies.

### 2.6 Practical Implications: Scalability, Robustness, and Deployment

Beyond cooling performance, the proposed film platform offers clear advantages for practical greenhouse deployment. As shown in Figure 1e, mechanical testing shows that the TiO_2_–PET‐based films exhibit tensile strengths of 69–92 MPa, significantly exceeding those of conventional LDPE (∼17 MPa) and DCFs (∼16 MPa), showing the potential to serve as a durable primary greenhouse covering under harsh outdoor conditions. By integrating both cooling functionality and mechanical robustness within a single polymer laminate fabricated via scalable lamination processes, effective greenhouse cooling can be achieved without shading structures or added system complexity. Collectively, these results establish a simple, durable, and readily deployable pathway for mitigating heat stress in greenhouse agriculture under tropical and subtropical climates.

## 3 Conclusion

This work demonstrates that effective greenhouse cooling in tropical climates can be achieved by managing, rather than avoiding, the transparency–cooling trade‐off. Under high solar irradiance, some reduction in PAR is unavoidable; however, we show that this reduction can be engineered strategically to deliver measurable thermal benefits without compromising plant‐compatible illumination. The proposed multilayer films achieve daytime canopy‐level temperature reductions of ≈3–5°C under real‐world outdoor tropical conditions while operating at controlled PAR transmittance levels (∼57% and ∼37%) tailored to different crop tolerances. Rather than uniformly blocking light, cooling is realized by combining diffuse PAR transmission, NIR heat/UV rejection, and high MIR emissivity to enable continuous radiative heat dissipation. The PET‐based laminate provides sufficient mechanical robustness to function as a durable primary greenhouse covering. In the future, spectral‐selective greenhouse covers may enable climate‐adaptive cultivation in hot regions, reducing cooling energy demand while maintaining crop productivity, and offering a passive pathway toward resilient and low‐carbon food production.

## 4 Method Section

### 4.1 Fabrication of TiO_2_‐Embedded PET Film

Commercial‐grade PET pellets were used as the polymer matrix. Titanium dioxide (TiO_2_, rutile phase; nominal primary particle size ∼200 nm) was incorporated as the filler. The particle size distribution after processing was characterized by laser scattering, yielding a median size (D50) of ∼2.15 µm (Figure S6). To ensure uniform dispersion, TiO_2_ powder was first ball‐milled to reduce agglomeration, then dispersed in ethanol, and mixed thoroughly with PET pellets to promote surface wetting and particle adhesion. The mixture was dried at 100°C overnight to remove residual solvent and moisture, yielding TiO_2_‐coated PET pellets. Composite batches containing 0.1–5 wt% TiO_2_ were prepared. A UV stabilizer was added to all formulations to improve outdoor durability. The dried pellets were processed using a single‐screw extruder to produce composite sheets with an average thickness of ≈70 µm. The 0.5 wt% TiO_2_–PET film was selected for further characterization following preliminary optical and thermal screening. Two independently fabricated batches (denoted as Lot 1 and Lot 2) were evaluated to confirm reproducibility, showing consistent optical and thermal behavior (see Figures S1–S3).

To impart spectral selectivity, a commercial UV–IR‐cut window film (automotive grade) was laminated onto the TiO_2_–PET surface without thermal bonding. A fine mist of distilled water was introduced at the interface to improve adhesion and prevent trapped air. This water‐assisted lamination produced a smooth, defect‐free multilayer structure that preserved visible transparency, introduced strong NIR rejection, and retained the intrinsically high MIR emissivity of the UV–IR film, properties beneficial for passive greenhouse cooling.

### 4.2 Film Design Strategy

The multilayer cooling film was designed using a spectral‐engineering framework targeting the two dominant radiative pathways governing greenhouse thermal balance: solar NIR heat gain and thermal MIR heat dissipation. TiO_2_ nanoparticles were embedded in the PET matrix to introduce controlled visible–NIR scattering and partial UV attenuation. Owing to the high refractive‐index contrast (*n* ≈ 2.7), the embedded particles diffuse incident sunlight and reduce effective solar heat input while maintaining a functional PAR window for crop growth. To further regulate solar heat gain and thermal emission, a commercial UV–IR window film containing nanometric multilayer interference structures was laminated onto the TiO_2_–PET substrate. The UV–IR selective layer was oriented toward the sky‐facing side of the multilayer structure. The UV–IR film intrinsically exhibits high MIR absorptivity and emissivity while strongly suppressing NIR transmission. When integrated into the laminated structure, the UV–IR layer retains its high emissivity and complements the scattering‐induced solar‐band control of the TiO_2_–PET substrate. The resulting multilayer architecture yields a combined spectral response characterized by reduced NIR heat input, diffused PAR delivery, and efficient thermal emission through the atmospheric transparency window. Two UV–IR variants were selected to span the lighting and thermal requirements of different crop types. UV–IR 70 provides higher visible transmittance suitable for light‐demanding crops, whereas UV–IR 45 offers stronger NIR suppression and moderated PAR transmission appropriate for heat‐sensitive and shade‐tolerant crops.

### 4.3 Structural and Compositional Characterization

The internal morphology of the TiO_2_–PET composite films was examined using cross‐sectional SEM (JEOL JSM‐IT300, 20 kV accelerating voltage). Film samples were cryo‐fractured in liquid nitrogen to expose a clean cross‐section and subsequently sputter‐coated with a thin platinum layer to minimize surface charging during imaging. SEM micrographs were acquired at 500× magnification to assess the dispersion of TiO_2_ particles and the interfacial compatibility between the filler and the PET matrix. The analysis was conducted to qualitatively assess particle dispersion and interfacial morphology, rather than to provide quantitative particle size distribution. Elemental composition and spatial distribution were evaluated using energy‐dispersive X‐ray spectroscopy operated in mapping mode. Elemental maps of titanium (Ti) and oxygen (O) were collected across the film cross‐section to verify the uniform dispersion of TiO_2_ within the polymer phase. The datasets were processed using JEOL Analysis Station software to overlay Ti and O signals onto the SEM images, enabling direct visualization of particle distribution.

### 4.4 Optical Characterization

The optical properties of the films were characterized across the solar and MIR spectral regions. UV–Vis–NIR direct transmittance and reflectance spectra in the 300–2500 nm range were measured using a UV–Vis–NIR spectrophotometer (Hitachi UH1450) under normal incidence. PAR (400–700 nm) transmittance and NIR (700–2500 nm) reflectance were obtained by numerical integration of the measured spectra after baseline correction.

MIR hemispherical reflectance (3–16 µm) was measured using a Fourier transform infrared spectrometer (Nicolet APEX) equipped with a gold‐coated integrating sphere (PIKE 660–108 200). The spectral emissivity, ε(λ), was calculated as ε(λ) = 1 − R(λ), where R(λ) is the measured hemispherical reflectance.

The blackbody‐weighted average emissivity over the atmospheric transparency window (8–13 μm) is defined as:

$${¯\text{ε}}_{8-13\;\text{µ}m}\left(T\right)=\frac{\int_{8\text{µ}m}^{13\text{µ}m}\text{ε}\left(\text{λ}\right)\;I_{\mathrm{BB}}\left(\text{λ},T\right)\;d\text{λ}}{\int_{8\text{µ}m}^{13\text{µ}m}I_{\mathrm{BB}}\left(\text{λ},T\right)d\text{λ}}$$

where $I_{\mathrm{BB}}(\lambda ,T)$is the spectral radiance of a blackbody at temperature T( taken as 300 K in this study).

### 4.5 Mechanical Characterization

The tensile properties of the films were characterized in accordance with ASTM D882 for thin plastic films. Rectangular specimens with a width of 1 inch (25.4 mm) and an overall length of 10 cm were prepared. Film thickness was measured at multiple positions using a digital micrometer, and the average thickness was used for stress calculations. Tensile tests were conducted at room temperature using a universal testing machine equipped with an appropriate load cell. The initial gauge length was set according to the specimen geometry, and a constant crosshead speed was applied in accordance with ASTM D882. For each film type, at least five specimens were tested to ensure reproducibility. The reported mechanical properties represent mean values with standard deviations.

### 4.6 Radiative Cooling Performance Evaluation (Foam Box)

Outdoor measurements were conducted on the rooftop of the Faculty of Science, Mahidol University (13.8° N, 100.3° E) on selected days during the tropical wet (monsoon) season (19 July, 24^th^ July, and 2^nd^ August 2025) under predominantly clear‐sky conditions. Measurements were performed between 11:00 and 14:00 local time, corresponding to peak solar conditions. A custom‐built outdoor testing apparatus was designed to minimize conductive and convective heat loss while emphasizing radiative heat exchange at the exposed surface. Each unit consisted of an insulated foam box lined with aluminum foil to suppress lateral heat exchange. A 10 × 10 cm opening was cut at the top of each box, where the test film was tightly mounted and sealed to form the exposed surface (Figure 3). Prior to measurement, all testing units were calibrated to ensure consistent thermal response across test units (Figure S3). Temperature measurements were obtained using Type‐K thermocouples connected to a digital data logger. Sensors were positioned directly beneath the center of the film and within the air cavity of each box. Ambient air temperature and incident solar irradiance were recorded simultaneously for reference.

### 4.7 Solar Irradiance Measurement Under Controlled 1 Sun Illumination

Transmitted irradiance measurements were conducted using an AAA‐class LED solar simulator (Ossila) as the irradiation source. The incident irradiance was calibrated to 1 Sun (1000 W m^−2^). A silicon photodiode detector (Model 91 150V, Newport) was used to measure transmitted irradiance at a fixed position beneath the samples.

### 4.8 Outdoor Solar Irradiance Measurement

Outdoor solar irradiance was measured using a silicon photodiode detector (Model 91 150V, Newport) under natural sunlight. Measurements were conducted near midday under stable conditions, with solar irradiance of ≈720–730 W m^−2^ during the measurement period. The detector was positioned at plant canopy level, and each value represents an average over a short time interval. All measurements were conducted sequentially with a fixed detector position. The measured signal from each sample was normalized to the incident solar intensity (Sun) measured under identical conditions. The normalized transmittance was calculated as the ratio of the transmitted signal through each sample to the corresponding incident solar signal. Reported values represent the average of 10 repeated measurements and are expressed as normalized values relative to the incident solar intensity.

### 4.9 Small‐Scale Greenhouse Construction and Testing

Small‐scale greenhouse units were constructed to evaluate the thermal performance of different roof covering configurations. Each unit consists of a frame assembled from ½‐inch diameter PVC pipes. LDPE film or test films were used as the covering materials and secured to the frame using standard greenhouse clips. All units were fabricated with identical geometry and assembly procedures to ensure consistency across samples. The evaluated roof configurations include pristine LDPE, TiO_2_–PET/UV–IR 45, TiO_2_–PET/UV–IR 70, and LDPE combined with a commercial shade net (60% shading indicated by the manufacturer). The greenhouse units were installed on the rooftop of the Faculty of Science, Mahidol University, under unobstructed sunlight and arranged to minimize mutual shading. Temperature was monitored using thermocouples positioned at the plant canopy level as shown in Figure S10. For the comparative dataset presented in Figure 4, all greenhouse configurations were evaluated on the same day under identical outdoor conditions (11:00–14:00, hot season April 19, 2026, clear‐sky).

## Author Contributions

**Pharit Gridtayawong**: Conceptualization, methodology, validation, formal analysis, investigation, writing – original draft, writing – review and editing, visualization. **Taweesak Kaewmanee**: methodology, validation, formal analysis, investigation. **Wachara Benchaphanthawee**: validation, writing – review and editing. **Varakorn Phiriyasas**: investigation. **Chattrarat Ponghiransmith**: methodology, investigation. **Worawut Rueangsawang**: methodology, investigation. **Chaowaphat Seriwattanachai**: writing – review and editing. **Patawee Sakata**: investigation. **Napong Tangwiroon**: investigation. **Thantham Jittham**: investigation. **Napan Phuphathanaphong**: investigation. **Phatratorn Wonganannont**: investigation. **Tansuda Pinpapat**: investigation. **Tanant Waritanant**: resources. **Tatpong Tulyananda**: resources. **Pongsakorn Kanjanaboos**: Conceptualization, methodology: validation, formal analysis, resources, writing – original draft, writing – review and editing, visualization, supervision, project administration, funding acquisition.

## Funding

This work was supported by the National Research Council of Thailand (NRCT) with the grant number N42A680232. The first author has received funding support from the Scholarship for Young Scientists program from Faculty of Science, Mahidol University.

## Conflicts of Interest

The work is under patent filing process.

## Supporting information

Supplementary Material

## Data Availability

The data that support the findings of this study are available from the corresponding author upon reasonable request.
