# Supplementary material for: Enhancing the Transparency–Temperature Trade‐Off Through Spectral Engineering and Radiative Cooling
Source: Small Sci. 2026 May 28;6(6):e70311. doi: 10.1002/smsc.70311 (PMC13248842; doi:10.1002/smsc.70311)
Supplement: Supplementary file 1 — Supplementary Material [file SMSC-6-e70311-s001.pdf]

Supporting Information

for

Enhancing the Transparency–Temperature Trade-Off through Spectral Engineering and Radiative Cooling

Pharit Gridtayawong[1, 2], Taweesak Kaewmanee[1, 2], Wachara Benchaphanthawee[1, 2], Varakorn Phiriyasas[1], Chattrarat Ponghiransmith[1, 2], Worawut Rueangsawang[1, 2], Chaowaphat Seriwattanachai[1], Patawee Sakata[1, 2], Napong Tangwiroon[1], Thantham Jittham[1], Napan Phuphathanaphong[1], Phatratorn Wongananonont[4], Tansuda Pinpapat[5], Tanant Waritanant [1], Tatpong Tulyananda[6], and Pongsakorn Kanjanaboos[1, 2, 3, *]

[1]*School of Materials Science and Innovation, Faculty of Science, Mahidol University, Nakhon Pathom 73170, Thailand*
[2]*Center for Cooling and Energy-saving Materials, Faculty of Science, Mahidol University, Nakhon Pathom 73170, Thailand*
[3]*Center of Excellence for Innovation in Chemistry (PERCH-CIC), Ministry of Higher Education, Science, Research and Innovation, Bangkok 10400, Thailand*
[4]*School of Bioresources and Environmental Biology, Faculty of Science, Mahidol University, Nakhon Pathom 73170, Thailand*
[5]*Department of Environmental Science and Technology, Faculty of Environment and Resource Studies, Mahidol University, Nakhon Pathom 73170, Thailand*
[6]*School of Bioinnovation and Bio-based Product Intelligence, Faculty of Science, Mahidol University, Nakhon Pathom 73170, Thailand*

[*]E-mail address of corresponding author: pongsakorn.kan@mahidol.edu

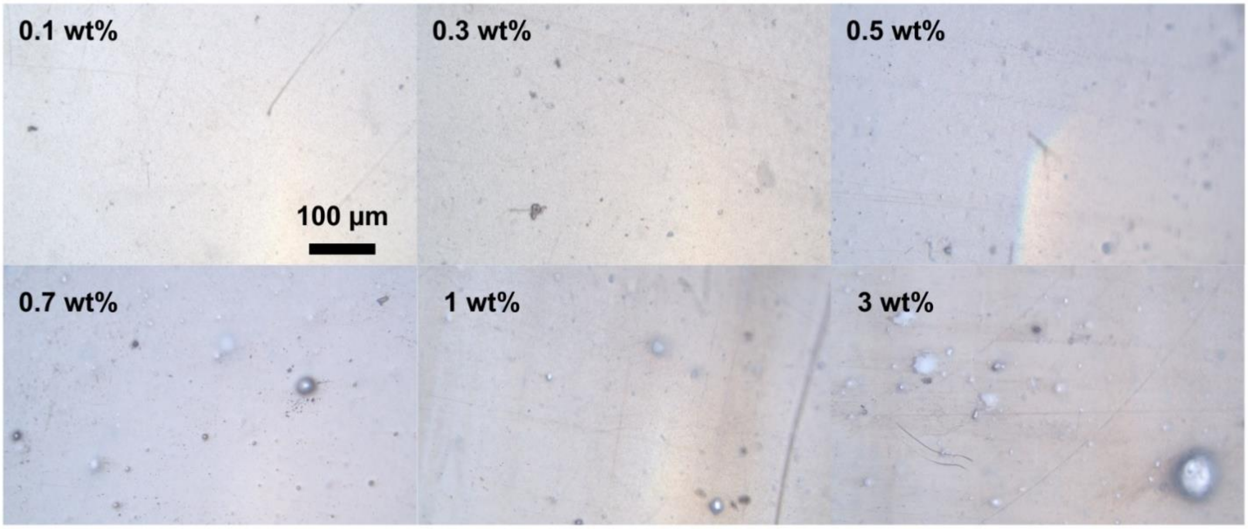

Figure S1. Optical microscopy images of TiO_2–PET films with varying TiO_2 concentrations (0.1–3 wt%). Scale bar: 100 µm.

Optical microscopy was employed to qualitatively assess particle dispersion of TiO_2–PET films across the investigated composition range. As the TiO_2 loading increases, particle agglomeration becomes more apparent.

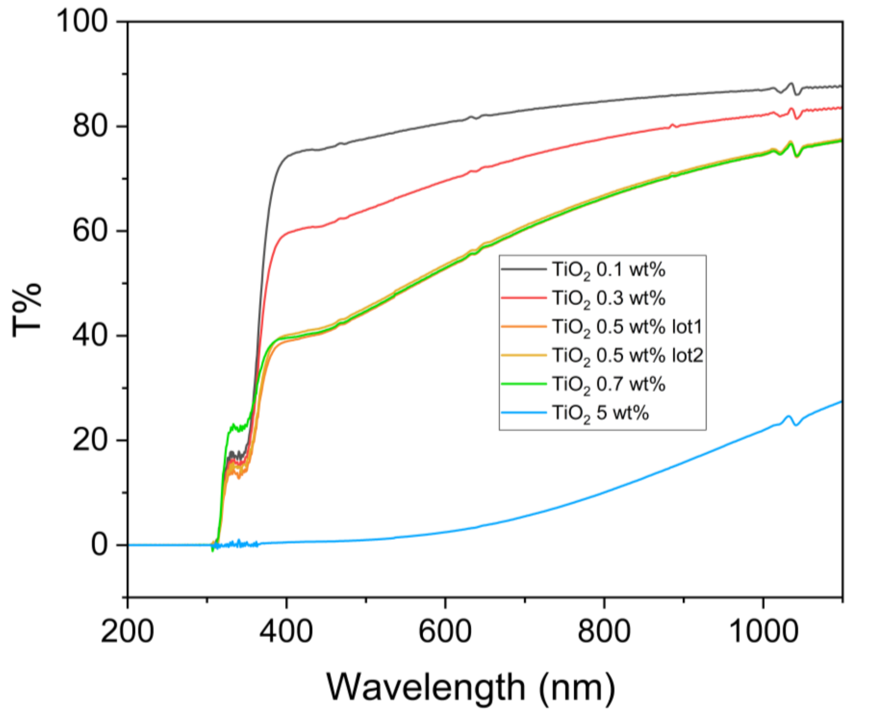

Figure S2. UV–Vis–NIR transmittance spectra of selected TiO_2–PET films at representative TiO_2 loadings.

UV–Vis transmittance measurements were conducted on representative compositions to capture overall optical trends. Increasing TiO_2 loading results in a systematic reduction in optical transmittance across the visible and near-infrared regions, consistent with enhanced scattering from TiO_2 inclusions. For the 0.5 wt% composition, two independently fabricated batches (denoted as Lot 1 and Lot 2) are included to demonstrate sample-to-sample reproducibility. The nearly identical spectral responses confirm consistent optical behavior and reliable film fabrication.

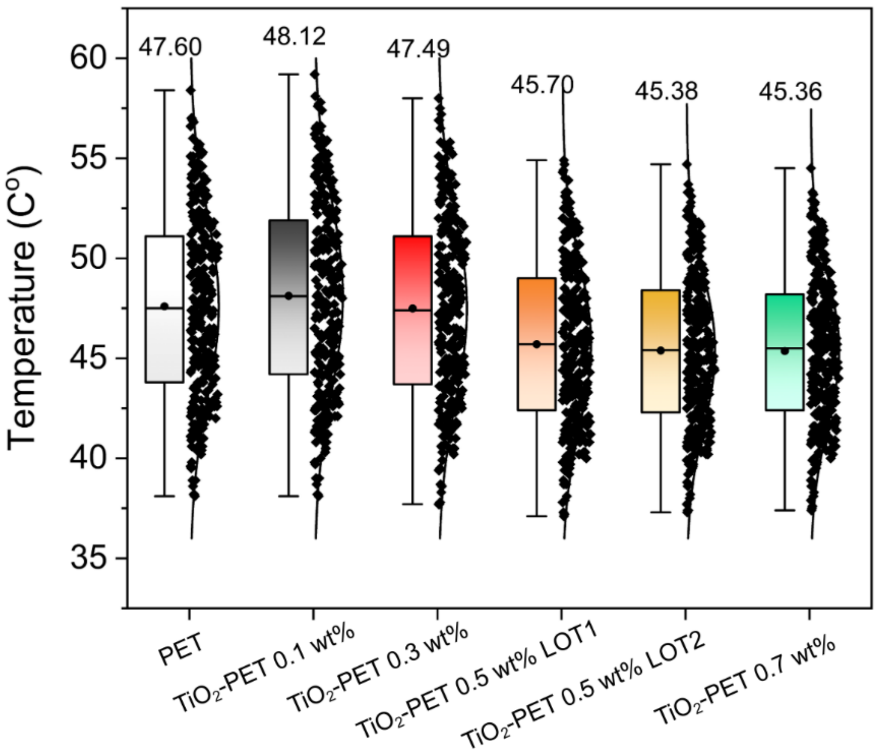

Figure S3. Statistical distribution of daytime temperatures measured for PET and selected TiO_2–PET films under outdoor rooftop conditions. Data points represent time-resolved temperature measurements, while box plots indicate median values and interquartile ranges.

Data points represent the statistical distributions of daytime temperatures measured for selected TiO_2–PET films under outdoor rooftop conditions. Although the 0.7 wt% TiO_2 film exhibits a comparable temperature reduction to the 0.5 wt% sample, increasing TiO_2 loading leads to more pronounced particle agglomeration, as observed in optical microscopy images (Fig. S1). This agglomeration reduces film optical uniformity without providing a substantial additional cooling benefit. To assess reproducibility, two independently fabricated 0.5 wt% TiO_2–PET samples (Lot 1 and Lot 2) were evaluated and show consistent temperature distributions. This agreement confirms the fabrication process and supports the selection of 0.5 wt% TiO_2 as a practical composition balancing optical performance and thermal regulation.

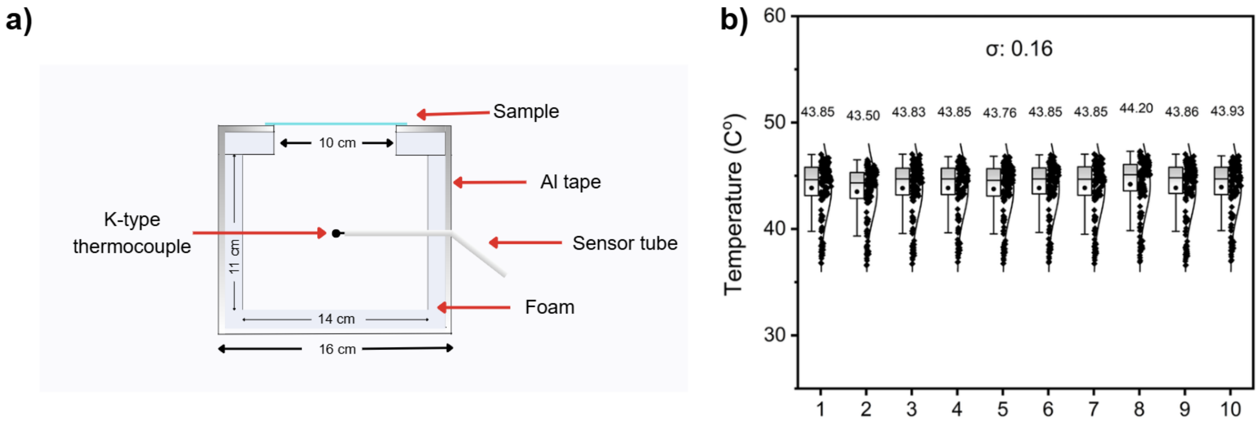

Figure S4. Closed-box calibration and measurement consistency. (a) Schematic of the insulated closed-box setup. The inner cavity measures 14 × 15 × 11 cm (width × length × height), corresponding to a projected ground area of ~0.021 m^2. The sample was mounted over a 10 × 10 cm top aperture (~0.010 m^2) and sealed with tape to prevent air leakage. A K-type thermocouple is positioned at the cavity center to record internal air temperature. (b) Temperature distributions from ten identical units under identical outdoor conditions using the same reference film. Box plots show the median and interquartile range, with scatter points indicating individual readings. The low standard deviation ($\sigma \approx 0.16$ °C) confirms high measurement consistency across units.

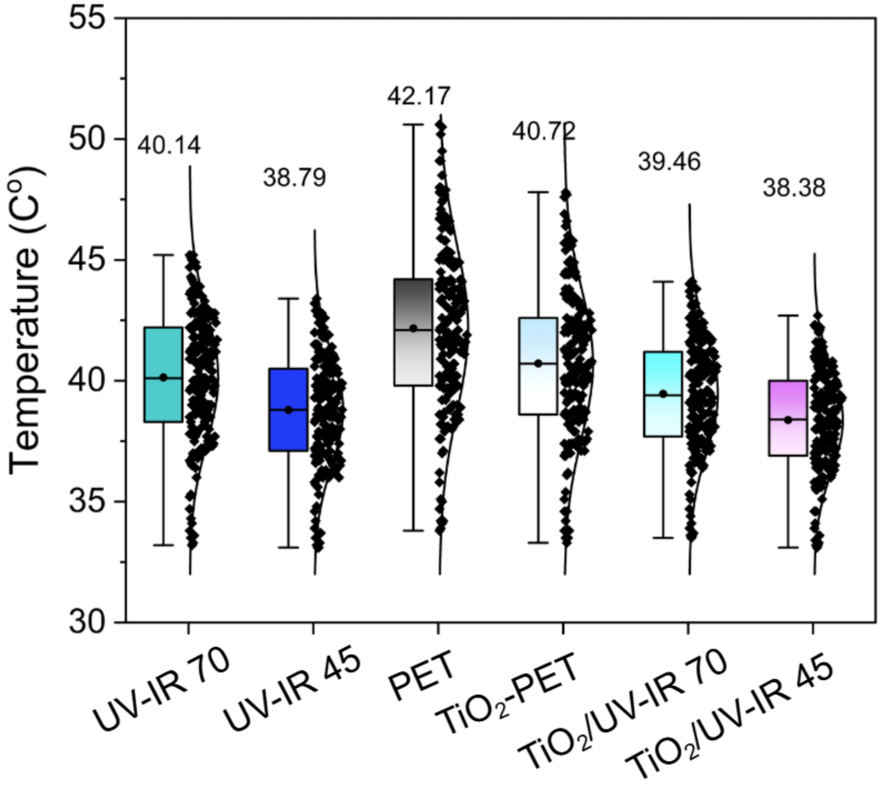

Figure S5. Distribution of measured daytime temperatures for different films measured simultaneously under identical outdoor conditions during 11:00–14:00. Individual data points represent time-resolved temperature measurements, while box plots indicate the median values and interquartile ranges. The TiO_2/UV–IR 45 film exhibits the lowest median daytime temperature among the evaluated samples, indicating the most effective cooling performance.

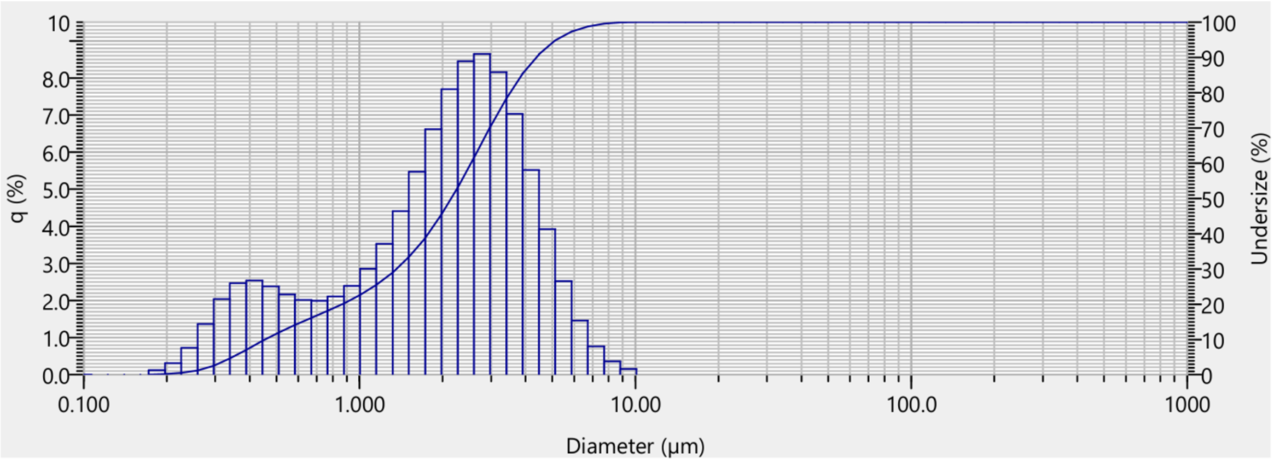

Figure S6. Particle size distribution of rutile TiO_2 particles measured by laser scattering (HORIBA LA-350). The distribution is presented on a volume basis, and the reported particle size corresponds to the equivalent spherical diameter. Here, D_x represents the particle diameter below which x% of the cumulative particle volume is contained. The median particle size (D50) is approximately 2.15 μm, with D10 ≈ 0.46 μm and D90 ≈ 4.38 μm, indicating a broad micron-scale distribution.

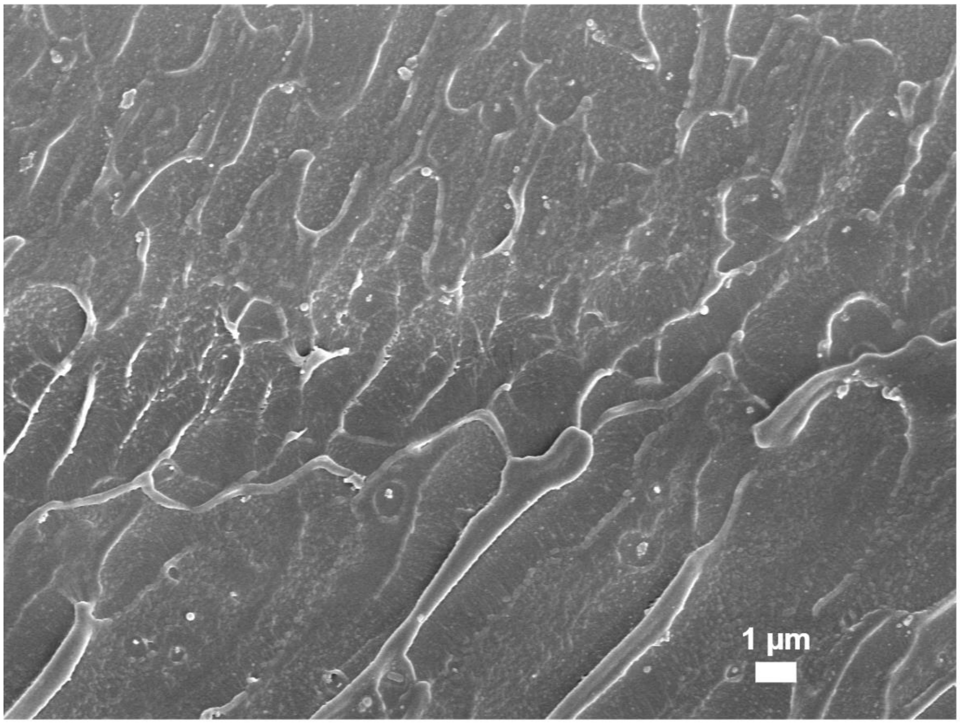

Figure S7. SEM image of the cryo-fractured cross-section of the TiO_2–PET composite film (×5000). The microstructure appears relatively homogeneous at this scale, with TiO_2 domains embedded within the PET matrix and limited contrast between phases. As a result, individual particles cannot be reliably distinguished, precluding direct particle size quantification from SEM imaging. This observation suggests that TiO_2 exists as dispersed and partially aggregated domains within the polymer matrix. Therefore, particle size distribution was evaluated using laser scattering measurements (Figure S6), which provide statistically representative values.

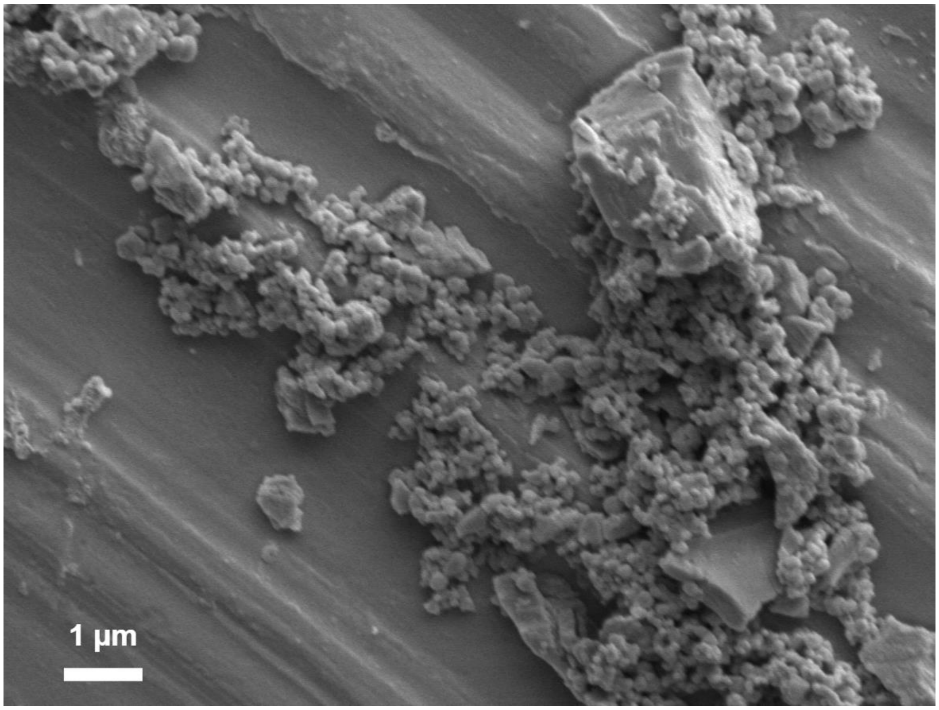

Figure S8. SEM image of as-received rutile TiO_2 particles deposited on a copper substrate (×10,000). The particles consist of fine primary domains that form aggregated clusters with irregular morphology. While the primary particle size is in the submicron range, significant agglomeration leads to the formation of micron-scale domains. This aggregation behavior is consistent with the particle size distribution obtained by laser scattering (Figure S6) and explains the effective scattering features observed in the composite films. In particular, the resulting size regime is consistent with Mie scattering across the visible–NIR range, supporting the observed broadband light redistribution in the composite films.

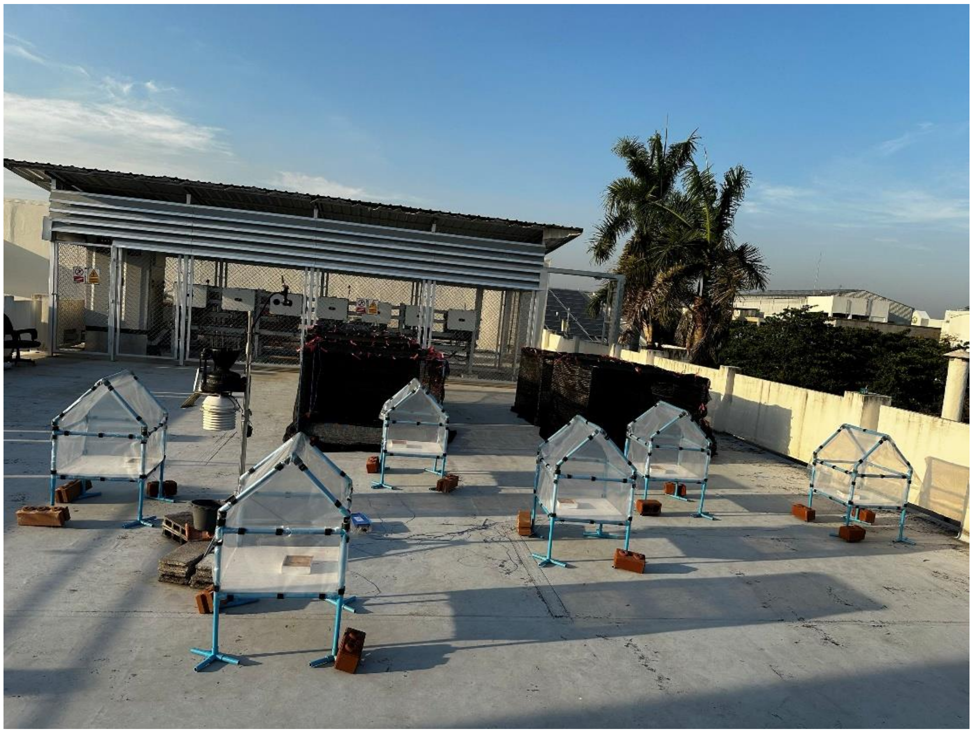

Figure S9. Small-scale greenhouse experimental setup for outdoor validation. Multiple identical PVC-frame greenhouses were constructed and placed on a rooftop under unobstructed solar exposure. All structures were enclosed using LDPE. The layout ensures comparable environmental exposure across units, enabling controlled evaluation of thermal performance under realistic conditions.

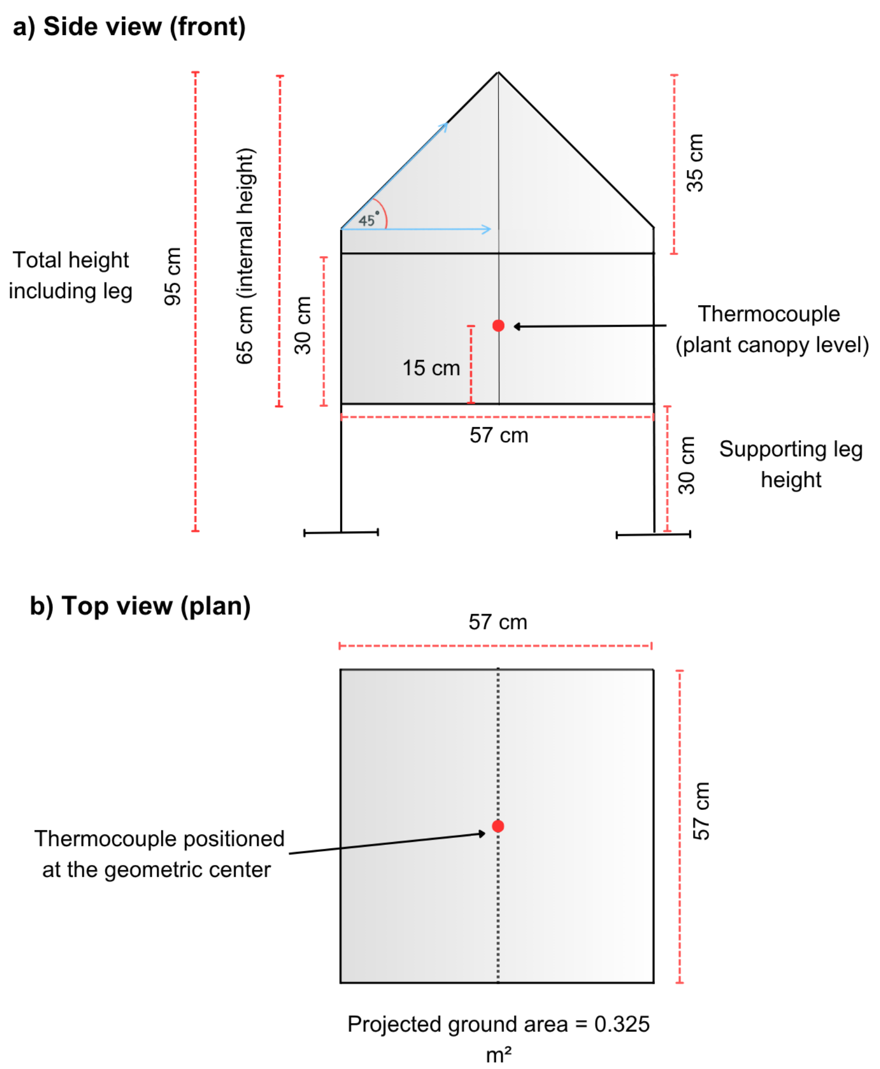

Figure S10: Schematic diagram of the customized small-scale greenhouse used for thermal performance testing. (a) Side view showing the internal dimensions and the placement of the K-type thermocouple. The sensor tip was suspended in mid-air at the plant canopy level (15 cm above the base) to measure the ambient air temperature without conductive interference from the structure. (b) Top view showing the thermocouple's horizontal alignment at the geometric center (0.325 m^2 projected area) to minimize boundary effects and ensure uniform measurement of the controlled environment under the experimental greenhouse films.

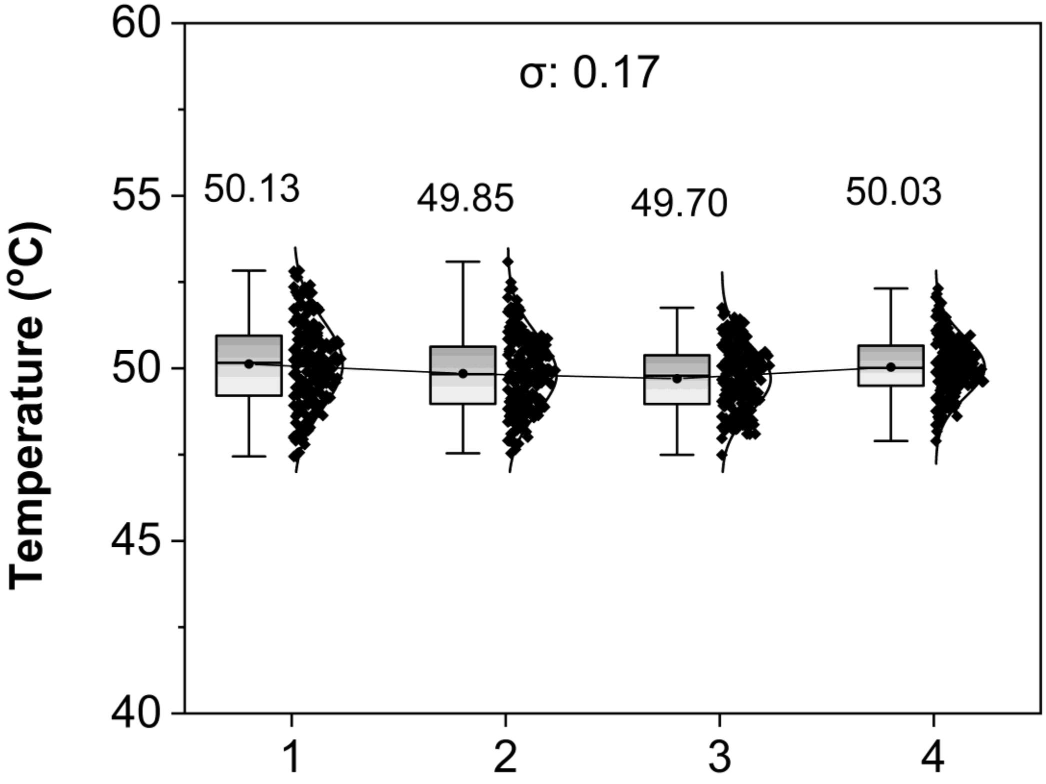

Figure S11. Temperature distributions measured from four identical closed test units under identical outdoor conditions during calibration. Data were collected during the peak solar period (11:00–14:00) and are presented as box–violin plots. The mean temperatures (49.70–50.13 °C) and the low standard deviation (σ = 0.17 °C) confirm the high consistency of the test units prior to comparative measurements.

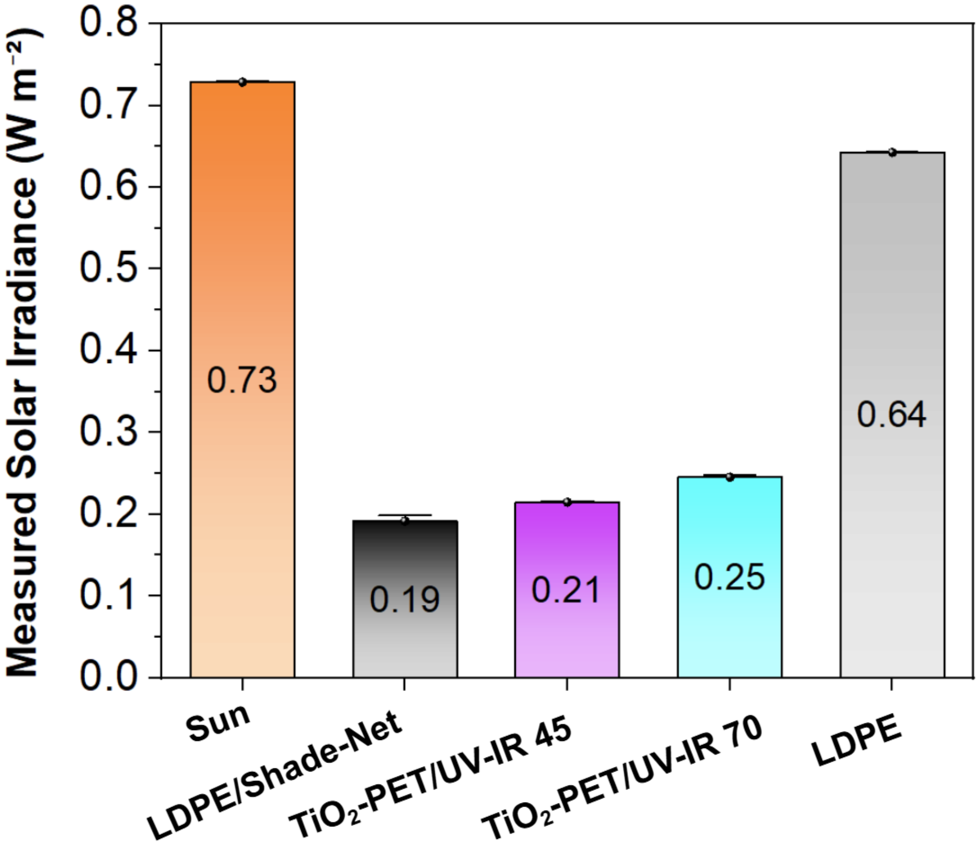

Figure S12. Measured transmitted solar irradiance under different greenhouse covering configurations under natural sunlight with normalization respective to the actual solar irradiation (clear-sky condition, 720-730 W m^{-2}). Measurements were conducted using a silicon photodiode detector (Newport 91150V) positioned at plant canopy level. Values represent the raw detected irradiance prior to normalization to the incident solar intensity used in Figure 4d.

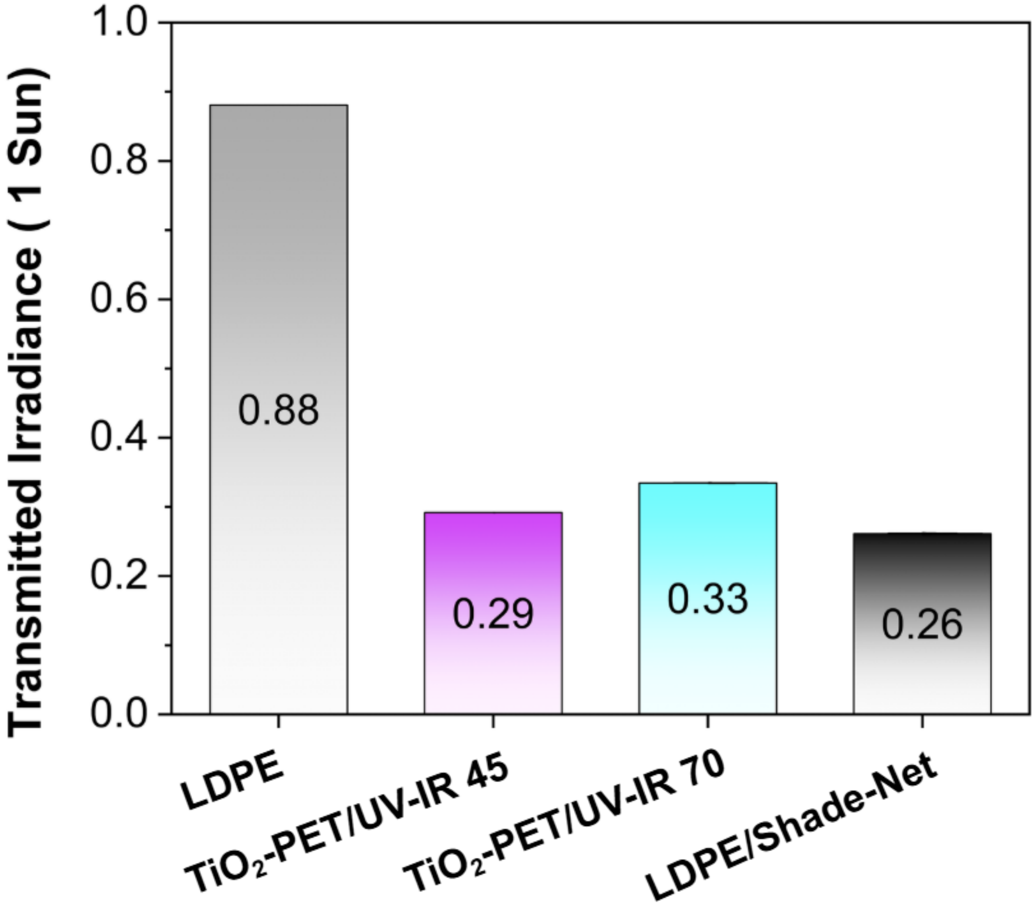

Figure S13. Transmitted solar irradiance of different greenhouse covering configurations under controlled 1 Sun illumination (indoor measurement). LDPE shows high transmittance (~0.88 Sun), whereas TiO_2–PET/UV–IR45 and TiO_2–PET/UV–IR 70 reduce transmitted irradiance to ~0.29 and ~0.34 Sun, respectively. The LDPE/shade-net configuration (~0.26 Sun) exhibits nearly identical transmitted irradiance to TiO_2–PET/UV–IR 45, confirming comparable solar loading.
